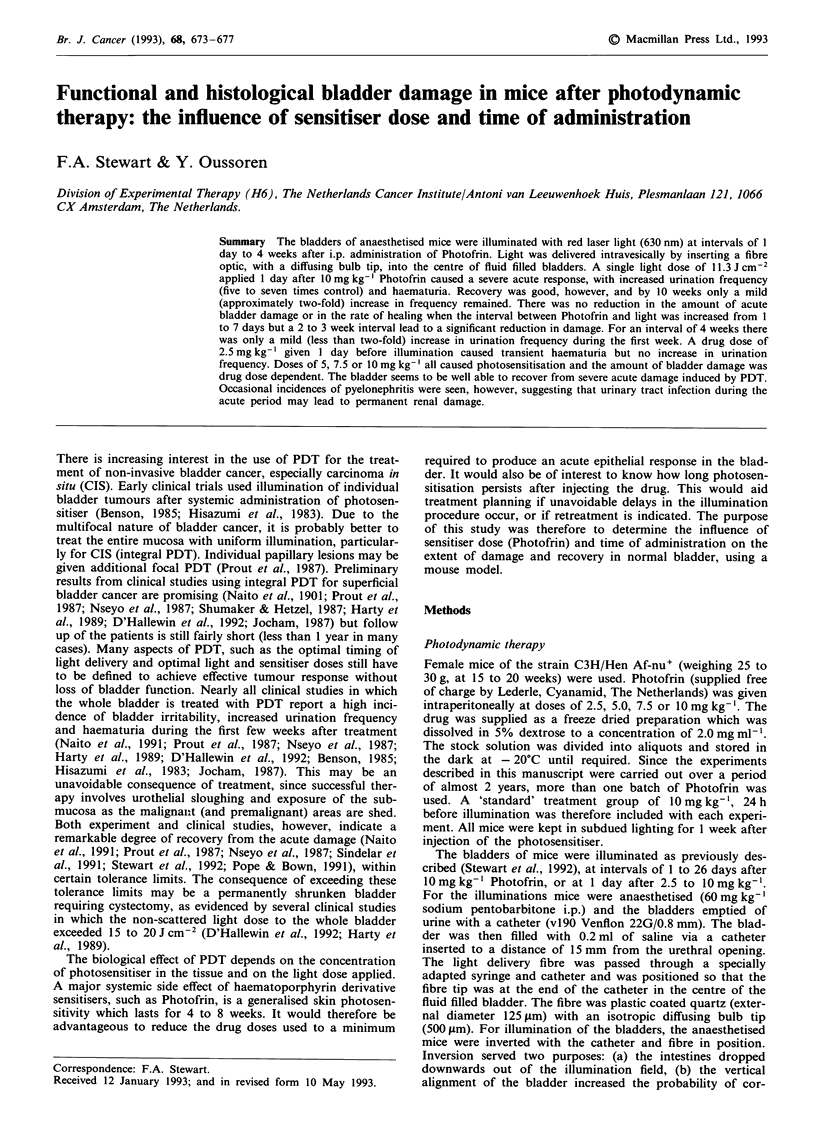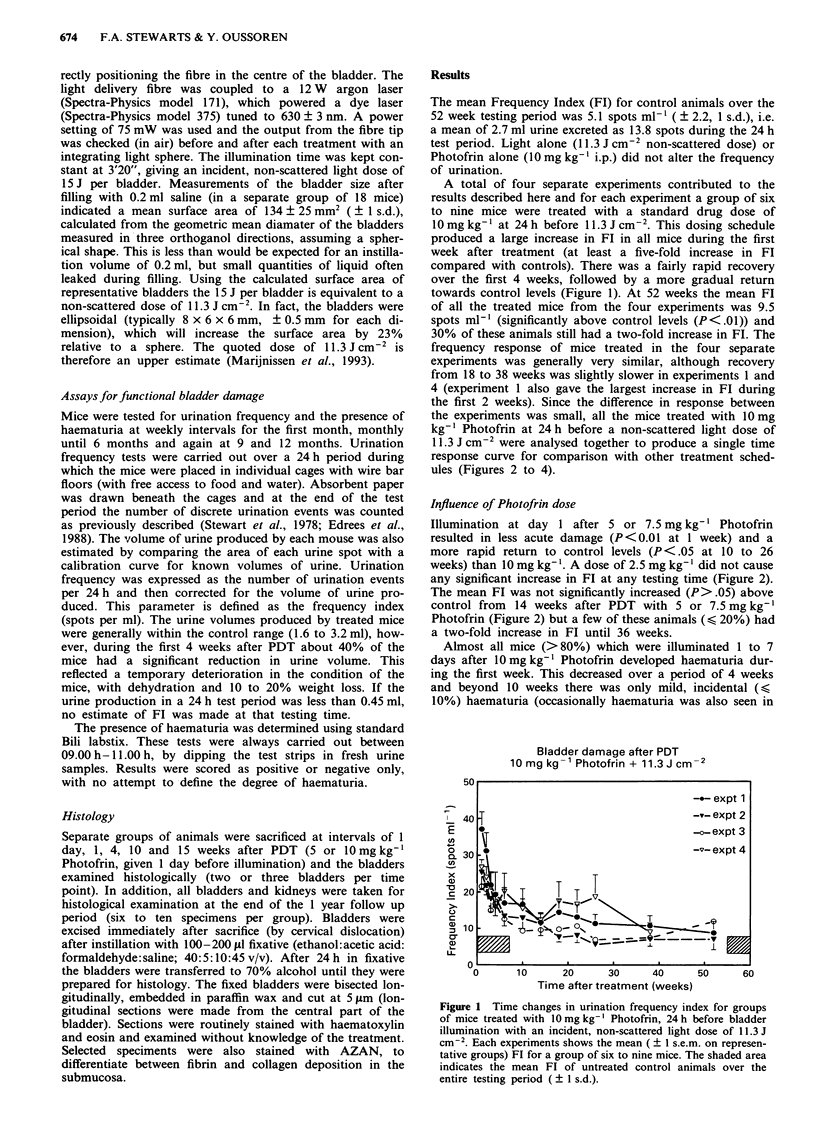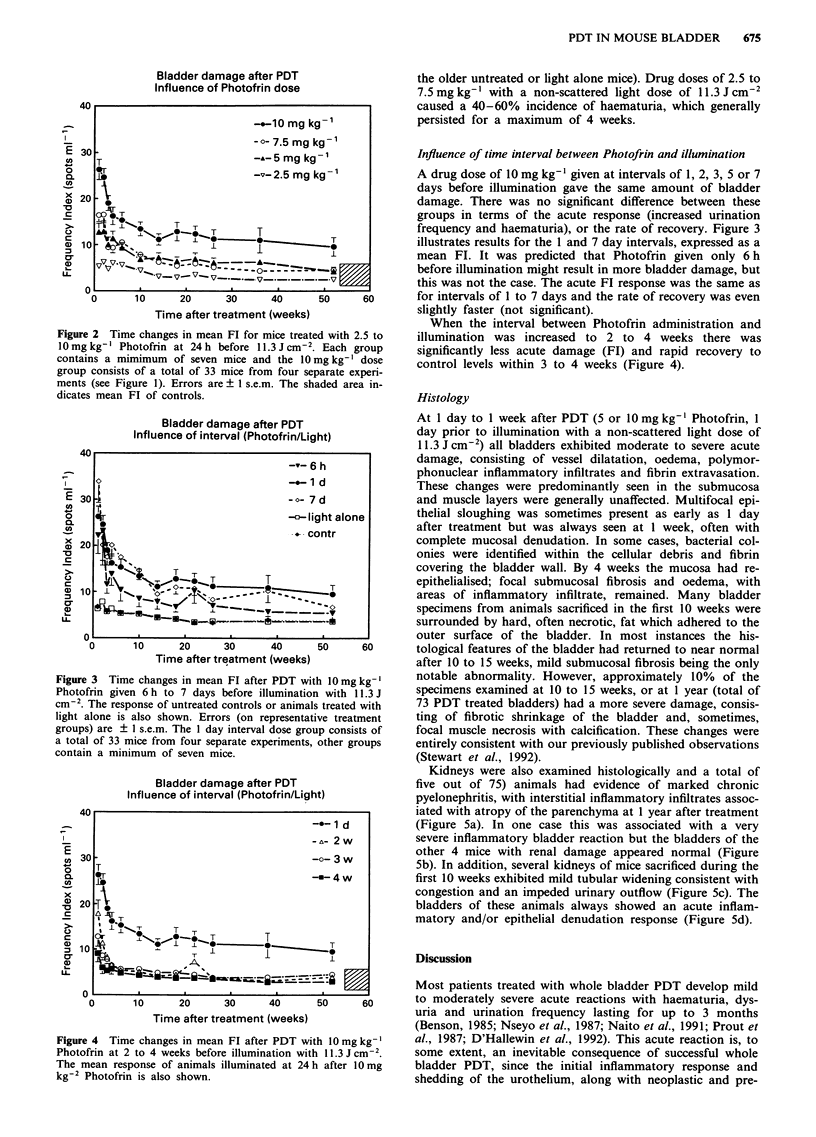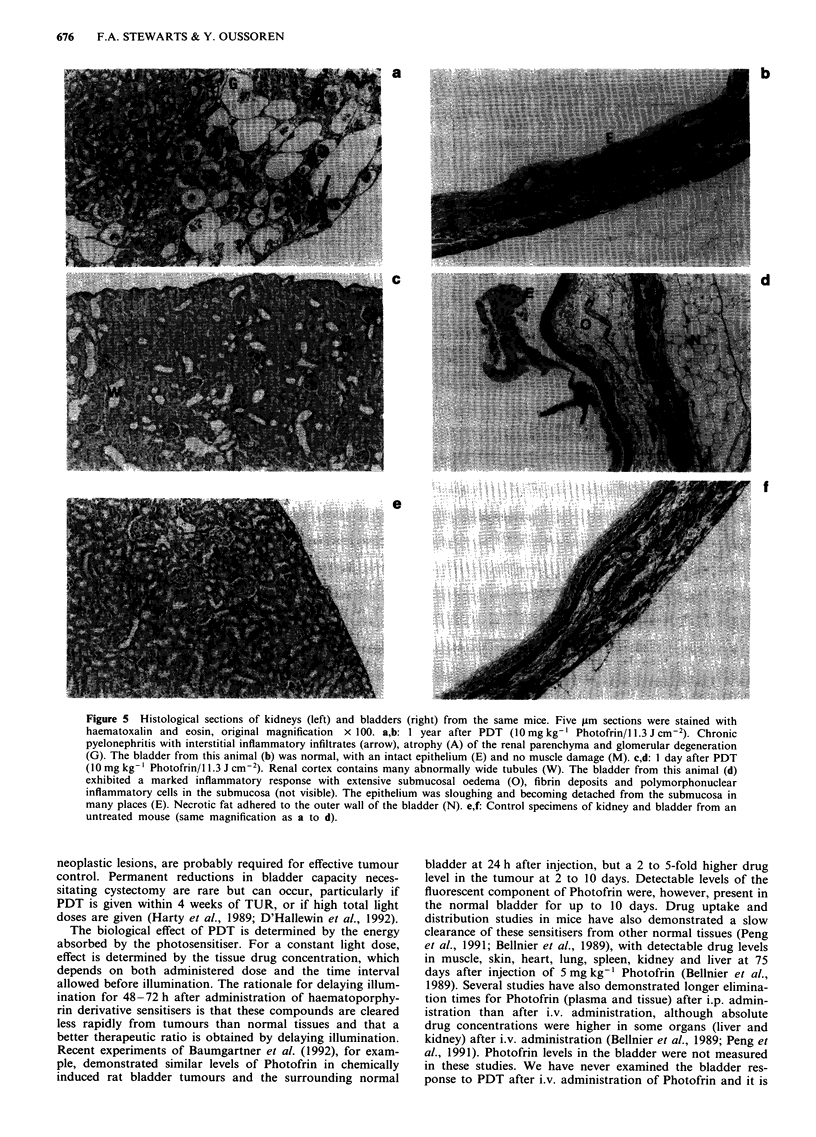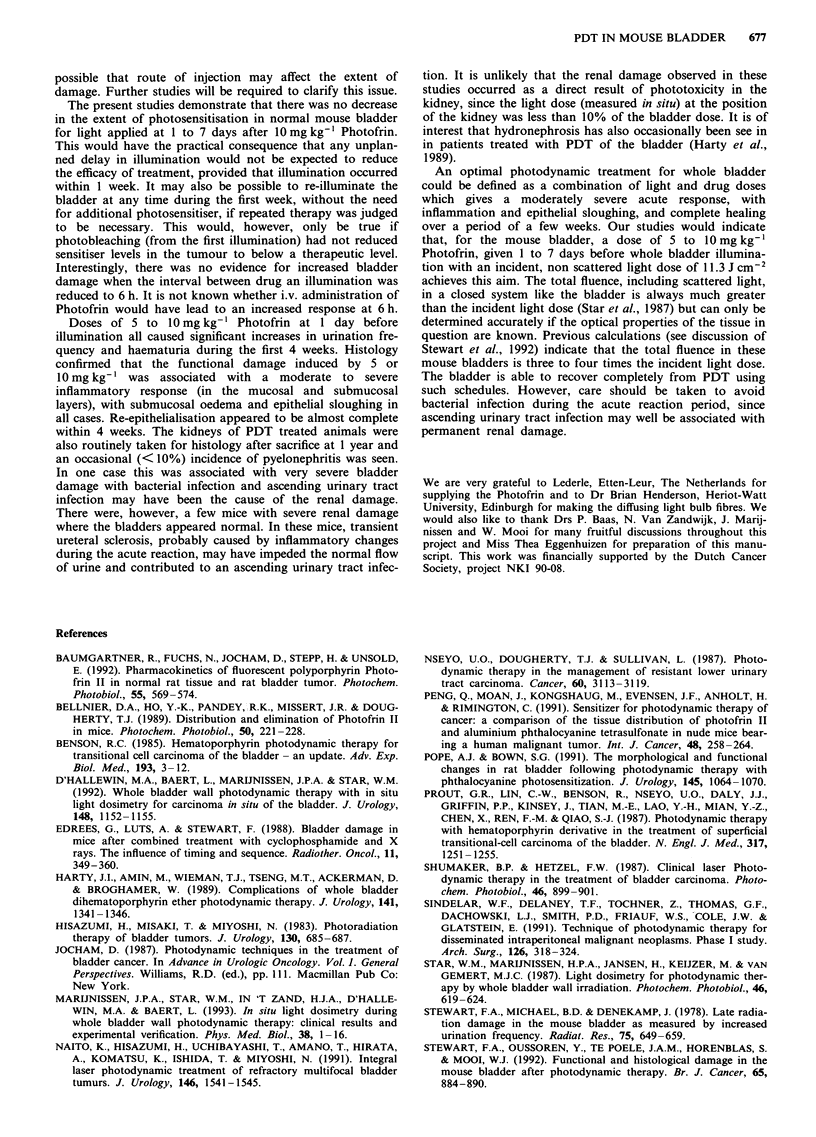# Functional and histological bladder damage in mice after photodynamic therapy: the influence of sensitiser dose and time of administration.

**DOI:** 10.1038/bjc.1993.407

**Published:** 1993-10

**Authors:** F. A. Stewart, Y. Oussoren

**Affiliations:** Division of Experimental Therapy, Netherlands Cancer Institute/Antoni van Leeuwenhoek Huis, Amsterdam.

## Abstract

**Images:**


Br. J. Cancer (1993), 68, 673-677 © Macmillan Press Ltd., 1993

### Functional and histological bladder damage in mice after photodynamic therapy: the influence of sensitiser dose and time of administration

F.A. Stewart & Y. Oussoren

*Division of Experimental Therapy (H6), The Netherlands Cancer Institute/Antoni van Leeuwenhoek Huis, Plesmanlaan 121, 1066 CX Amsterdam, The Netherlands.*

**Summary** The bladders of anaesthetised mice were illuminated with red laser light (630 nm) at intervals of 1 day to 4 weeks after i.p. administration of Photofrin. Light was delivered intravesically by inserting a fibre optic, with a diffusing bulb tip, into the centre of fluid filled bladders. A single light dose of 11.3 J $cm^{-2}$ applied 1 day after 10 mg $kg^{-1}$ Photofrin caused a severe acute response, with increased urination frequency (five to seven times control) and haematuria. Recovery was good, however, and by 10 weeks only a mild (approximately two-fold) increase in frequency remained. There was no reduction in the amount of acute bladder damage or in the rate of healing when the interval between Photofrin and light was increased from 1 to 7 days but a 2 to 3 week interval lead to a significant reduction in damage. For an interval of 4 weeks there was only a mild (less than two-fold) increase in urination frequency during the first week. A drug dose of 2.5 mg $kg^{-1}$ given 1 day before illumination caused transient haematuria but no increase in urination frequency. Doses of 5, 7.5 or 10 mg $kg^{-1}$ all caused photosensitisation and the amount of bladder damage was drug dose dependent. The bladder seems to be well able to recover from severe acute damage induced by PDT. Occasional incidences of pyelonephritis were seen, however, suggesting that urinary tract infection during the acute period may lead to permanent renal damage.

There is increasing interest in the use of PDT for the treatment of non-invasive bladder cancer, especially carcinoma *in situ* (CIS). Early clinical trials used illumination of individual bladder tumours after systemic administration of photosensitiser (Benson, 1985; Hisazumi *et al.*, 1983). Due to the multifocal nature of bladder cancer, it is probably better to treat the entire mucosa with uniform illumination, particularly for CIS (integral PDT). Individual papillary lesions may be given additional focal PDT (Prout *et al.*, 1987). Preliminary results from clinical studies using integral PDT for superficial bladder cancer are promising (Naito *et al.*, 1901; Prout *et al.*, 1987; Nseyo *et al.*, 1987; Shumaker & Hetzel, 1987; Harty *et al.*, 1989; D'Hallewin *et al.*, 1992; Jocham, 1987) but follow up of the patients is still fairly short (less than 1 year in many cases). Many aspects of PDT, such as the optimal timing of light delivery and optimal light and sensitiser doses still have to be defined to achieve effective tumour response without loss of bladder function. Nearly all clinical studies in which the whole bladder is treated with PDT report a high incidence of bladder irritability, increased urination frequency and haematuria during the first few weeks after treatment (Naito *et al.*, 1991; Prout *et al.*, 1987; Nseyo *et al.*, 1987; Harty *et al.*, 1989; D'Hallewin *et al.*, 1992; Benson, 1985; Hisazumi *et al.*, 1983; Jocham, 1987). This may be an unavoidable consequence of treatment, since successful therapy involves urothelial sloughing and exposure of the submucosa as the malignant (and premalignant) areas are shed. Both experiment and clinical studies, however, indicate a remarkable degree of recovery from the acute damage (Naito *et al.*, 1991; Prout *et al.*, 1987; Nseyo *et al.*, 1987; Sindelar *et al.*, 1991; Stewart *et al.*, 1992; Pope & Bown, 1991), within certain tolerance limits. The consequence of exceeding these tolerance limits may be a permanently shrunken bladder requiring cystectomy, as evidenced by several clinical studies in which the non-scattered light dose to the whole bladder exceeded 15 to 20 J $cm^{-2}$ (D'Hallewin *et al.*, 1992; Harty *et al.*, 1989).

The biological effect of PDT depends on the concentration of photosensitiser in the tissue and on the light dose applied. A major systemic side effect of haematoporphyrin derivative sensitisers, such as Photofrin, is a generalised skin photosensitivity which lasts for 4 to 8 weeks. It would therefore be advantageous to reduce the drug doses used to a minimum required to produce an acute epithelial response in the bladder. It would also be of interest to know how long photosensitisation persists after injecting the drug. This would aid treatment planning if unavoidable delays in the illumination procedure occur, or if retreatment is indicated. The purpose of this study was therefore to determine the influence of sensitiser dose (Photofrin) and time of administration on the extent of damage and recovery in normal bladder, using a mouse model.

### Methods

### *Photodynamic therapy*

Female mice of the strain C3H/Hen Af-$nu^+$ (weighing 25 to 30 g, at 15 to 20 weeks) were used. Photofrin (supplied free of charge by Lederle, Cyanamid, The Netherlands) was given intraperitoneally at doses of 2.5, 5.0, 7.5 or 10 mg $kg^{-1}$. The drug was supplied as a freeze dried preparation which was dissolved in 5% dextrose to a concentration of 2.0 mg $ml^{-1}$. The stock solution was divided into aliquots and stored in the dark at $-20$°C until required. Since the experiments described in this manuscript were carried out over a period of almost 2 years, more than one batch of Photofrin was used. A 'standard' treatment group of 10 mg $kg^{-1}$, 24 h before illumination was therefore included with each experiment. All mice were kept in subdued lighting for 1 week after injection of the photosensitiser.

The bladders of mice were illuminated as previously described (Stewart *et al.*, 1992), at intervals of 1 to 26 days after 10 mg $kg^{-1}$ Photofrin, or at 1 day after 2.5 to 10 mg $kg^{-1}$. For the illuminations mice were anaesthetised (60 mg $kg^{-1}$ sodium pentobarbitone i.p.) and the bladders emptied of urine with a catheter (v190 Venflon 22G/0.8 mm). The bladder was then filled with 0.2 ml of saline via a catheter inserted to a distance of 15 mm from the urethral opening. The light delivery fibre was passed through a specially adapted syringe and catheter and was positioned so that the fibre tip was at the end of the catheter in the centre of the fluid filled bladder. The fibre was plastic coated quartz (external diameter 125 μm) with an isotropic diffusing bulb tip (500 μm). For illumination of the bladders, the anaesthetised mice were inverted with the catheter and fibre in position. Inversion served two purposes: (a) the intestines dropped downwards out of the illumination field, (b) the vertical alignment of the bladder increased the probability of cor-

Correspondence: F.A. Stewart.
Received 12 January 1993; and in revised form 10 May 1993.

rectly positioning the fibre in the centre of the bladder. The light delivery fibre was coupled to a 12 W argon laser (Spectra-Physics model 171), which powered a dye laser (Spectra-Physics model 375) tuned to 630 ± 3 nm. A power setting of 75 mW was used and the output from the fibre tip was checked (in air) before and after each treatment with an integrating light sphere. The illumination time was kept constant at 3′20″, giving an incident, non-scattered light dose of 15 J per bladder. Measurements of the bladder size after filling with 0.2 ml saline (in a separate group of 18 mice) indicated a mean surface area of $134 \pm 25\ mm^2$ ( ± 1 s.d.), calculated from the geometric mean diamater of the bladders measured in three orthoganol directions, assuming a spherical shape. This is less than would be expected for an instillation volume of 0.2 ml, but small quantities of liquid often leaked during filling. Using the calculated surface area of representative bladders the 15 J per bladder is equivalent to a non-scattered dose of $11.3\ J\ cm^{-2}$. In fact, the bladders were ellipsoidal (typically 8 × 6 × 6 mm, ± 0.5 mm for each dimension), which will increase the surface area by 23% relative to a sphere. The quoted dose of $11.3\ J\ cm^{-2}$ is therefore an upper estimate (Marijnissen *et al.*, 1993).

### *Assays for functional bladder damage*

Mice were tested for urination frequency and the presence of haematuria at weekly intervals for the first month, monthly until 6 months and again at 9 and 12 months. Urination frequency tests were carried out over a 24 h period during which the mice were placed in individual cages with wire bar floors (with free access to food and water). Absorbent paper was drawn beneath the cages and at the end of the test period the number of discrete urination events was counted as previously described (Stewart *et al.*, 1978; Edrees *et al.*, 1988). The volume of urine produced by each mouse was also estimated by comparing the area of each urine spot with a calibration curve for known volumes of urine. Urination frequency was expressed as the number of urination events per 24 h and then corrected for the volume of urine produced. This parameter is defined as the frequency index (spots per ml). The urine volumes produced by treated mice were generally within the control range (1.6 to 3.2 ml), however, during the first 4 weeks after PDT about 40% of the mice had a significant reduction in urine volume. This reflected a temporary deterioration in the condition of the mice, with dehydration and 10 to 20% weight loss. If the urine production in a 24 h test period was less than 0.45 ml, no estimate of FI was made at that testing time.

The presence of haematuria was determined using standard Bili labstix. These tests were always carried out between 09.00 h–11.00 h, by dipping the test strips in fresh urine samples. Results were scored as positive or negative only, with no attempt to define the degree of haematuria.

### *Histology*

Separate groups of animals were sacrificed at intervals of 1 day, 1, 4, 10 and 15 weeks after PDT (5 or $10\ mg\ kg^{-1}$ Photofrin, given 1 day before illumination) and the bladders examined histologically (two or three bladders per time point). In addition, all bladders and kidneys were taken for histological examination at the end of the 1 year follow up period (six to ten specimens per group). Bladders were excised immediately after sacrifice (by cervical dislocation) after instillation with 100–200 µl fixative (ethanol:acetic acid: formaldehyde:saline; 40:5:10:45 v/v). After 24 h in fixative the bladders were transferred to 70% alcohol until they were prepared for histology. The fixed bladders were bisected longitudinally, embedded in paraffin wax and cut at 5 µm (longitudinal sections were made from the central part of the bladder). Sections were routinely stained with haematoxylin and eosin and examined without knowledge of the treatment. Selected speciments were also stained with AZAN, to differentiate between fibrin and collagen deposition in the submucosa.

### Results

The mean Frequency Index (FI) for control animals over the 52 week testing period was 5.1 spots $ml^{-1}$ ( ± 2.2, 1 s.d.), i.e. a mean of 2.7 ml urine excreted as 13.8 spots during the 24 h test period. Light alone ($11.3\ J\ cm^{-2}$ non-scattered dose) or Photofrin alone ($10\ mg\ kg^{-1}$ i.p.) did not alter the frequency of urination.

A total of four separate experiments contributed to the results described here and for each experiment a group of six to nine mice were treated with a standard drug dose of $10\ mg\ kg^{-1}$ at 24 h before $11.3\ J\ cm^{-2}$. This dosing schedule produced a large increase in FI in all mice during the first week after treatment (at least a five-fold increase in FI compared with controls). There was a fairly rapid recovery over the first 4 weeks, followed by a more gradual return towards control levels (Figure 1). At 52 weeks the mean FI of all the treated mice from the four experiments was 9.5 spots $ml^{-1}$ (significantly above control levels ($P < .01$)) and 30% of these animals still had a two-fold increase in FI. The frequency response of mice treated in the four separate experiments was generally very similar, although recovery from 18 to 38 weeks was slightly slower in experiments 1 and 4 (experiment 1 also gave the largest increase in FI during the first 2 weeks). Since the difference in response between the experiments was small, all the mice treated with 10 mg $kg^{-1}$ Photofrin at 24 h before a non-scattered light dose of $11.3\ J\ cm^{-2}$ were analysed together to produce a single time response curve for comparison with other treatment schedules (Figures 2 to 4).

### *Influence of Photofrin dose*

Illumination at day 1 after 5 or $7.5\ mg\ kg^{-1}$ Photofrin resulted in less acute damage ($P < 0.01$ at 1 week) and a more rapid return to control levels ($P < .05$ at 10 to 26 weeks) than $10\ mg\ kg^{-1}$. A dose of $2.5\ mg\ kg^{-1}$ did not cause any significant increase in FI at any testing time (Figure 2). The mean FI was not significantly increased ($P > .05$) above control from 14 weeks after PDT with 5 or $7.5\ mg\ kg^{-1}$ Photofrin (Figure 2) but a few of these animals (⩽ 20%) had a two-fold increase in FI until 36 weeks.

Almost all mice (> 80%) which were illuminated 1 to 7 days after $10\ mg\ kg^{-1}$ Photofrin developed haematuria during the first week. This decreased over a period of 4 weeks and beyond 10 weeks there was only mild, incidental (⩽ 10%) haematuria (occasionally haematuria was also seen in

**Figure 1** Time changes in urination frequency index for groups of mice treated with $10\ mg\ kg^{-1}$ Photofrin, 24 h before bladder illumination with an incident, non-scattered light dose of 11.3 J $cm^{-2}$. Each experiments shows the mean ( ± 1 s.e.m. on representative groups) FI for a group of six to nine mice. The shaded area indicates the mean FI of untreated control animals over the entire testing period ( ± 1 s.d.).

**Figure 2** Time changes in mean FI for mice treated with 2.5 to 10 mg kg$^{-1}$ Photofrin at 24 h before 11.3 J cm$^{-2}$. Each group contains a mimimum of seven mice and the 10 mg kg$^{-1}$ dose group consists of a total of 33 mice from four separate experiments (see Figure 1). Errors are ± 1 s.e.m. The shaded area indicates mean FI of controls.

**Figure 3** Time changes in mean FI after PDT with 10 mg kg$^{-1}$ Photofrin given 6 h to 7 days before illumination with 11.3 J cm$^{-2}$. The response of untreated controls or animals treated with light alone is also shown. Errors (on representative treatment groups) are ± 1 s.e.m. The 1 day interval dose group consists of a total of 33 mice from four separate experiments, other groups contain a minimum of seven mice.

**Figure 4** Time changes in mean FI after PDT with 10 mg kg$^{-1}$ Photofrin at 2 to 4 weeks before illumination with 11.3 J cm$^{-2}$. The mean response of animals illuminated at 24 h after 10 mg kg$^{-2}$ Photofrin is also shown.

the older untreated or light alone mice). Drug doses of 2.5 to 7.5 mg kg$^{-1}$ with a non-scattered light dose of 11.3 J cm$^{-2}$ caused a 40–60% incidence of haematuria, which generally persisted for a maximum of 4 weeks.

### *Influence of time interval between Photofrin and illumination*

A drug dose of 10 mg kg$^{-1}$ given at intervals of 1, 2, 3, 5 or 7 days before illumination gave the same amount of bladder damage. There was no significant difference between these groups in terms of the acute response (increased urination frequency and haematuria), or the rate of recovery. Figure 3 illustrates results for the 1 and 7 day intervals, expressed as a mean FI. It was predicted that Photofrin given only 6 h before illumination might result in more bladder damage, but this was not the case. The acute FI response was the same as for intervals of 1 to 7 days and the rate of recovery was even slightly faster (not significant).

When the interval between Photofrin administration and illumination was increased to 2 to 4 weeks there was significantly less acute damage (FI) and rapid recovery to control levels within 3 to 4 weeks (Figure 4).

### *Histology*

At 1 day to 1 week after PDT (5 or 10 mg kg$^{-1}$ Photofrin, 1 day prior to illumination with a non-scattered light dose of 11.3 J cm$^{-2}$) all bladders exhibited moderate to severe acute damage, consisting of vessel dilatation, oedema, polymorphonuclear inflammatory infiltrates and fibrin extravasation. These changes were predominantly seen in the submucosa and muscle layers were generally unaffected. Multifocal epithelial sloughing was sometimes present as early as 1 day after treatment but was always seen at 1 week, often with complete mucosal denudation. In some cases, bacterial colonies were identified within the cellular debris and fibrin covering the bladder wall. By 4 weeks the mucosa had re-epithelialised; focal submucosal fibrosis and oedema, with areas of inflammatory infiltrate, remained. Many bladder specimens from animals sacrificed in the first 10 weeks were surrounded by hard, often necrotic, fat which adhered to the outer surface of the bladder. In most instances the histological features of the bladder had returned to near normal after 10 to 15 weeks, mild submucosal fibrosis being the only notable abnormality. However, approximately 10% of the specimens examined at 10 to 15 weeks, or at 1 year (total of 73 PDT treated bladders) had a more severe damage, consisting of fibrotic shrinkage of the bladder and, sometimes, focal muscle necrosis with calcification. These changes were entirely consistent with our previously published observations (Stewart *et al.*, 1992).

Kidneys were also examined histologically and a total of five out of 75) animals had evidence of marked chronic pyelonephritis, with interstitial inflammatory infiltrates associated with atropy of the parenchyma at 1 year after treatment (Figure 5a). In one case this was associated with a very severe inflammatory bladder reaction but the bladders of the other 4 mice with renal damage appeared normal (Figure 5b). In addition, several kidneys of mice sacrificed during the first 10 weeks exhibited mild tubular widening consistent with congestion and an impeded urinary outflow (Figure 5c). The bladders of these animals always showed an acute inflammatory and/or epithelial denudation response (Figure 5d).

### Discussion

Most patients treated with whole bladder PDT develop mild to moderately severe acute reactions with haematuria, dysuria and urination frequency lasting for up to 3 months (Benson, 1985; Nseyo *et al.*, 1987; Naito *et al.*, 1991; Prout *et al.*, 1987; D'Hallewin *et al.*, 1992). This acute reaction is, to some extent, an inevitable consequence of successful whole bladder PDT, since the initial inflammatory response and shedding of the urothelium, along with neoplastic and pre-

**Figure 5** Histological sections of kidneys (left) and bladders (right) from the same mice. Five $\mu$m sections were stained with haematoxalin and eosin, original magnification $\times$ 100. **a,b**: 1 year after PDT (10 mg $kg^{-1}$ Photofrin/11.3 J $cm^{-2}$). Chronic pyelonephritis with interstitial inflammatory infiltrates (arrow), atrophy (A) of the renal parenchyma and glomerular degeneration (G). The bladder from this animal (**b**) was normal, with an intact epithelium (E) and no muscle damage (M). **c,d**: 1 day after PDT (10 mg $kg^{-1}$ Photofrin/11.3 J $cm^{-2}$). Renal cortex contains many abnormally wide tubules (W). The bladder from this animal (**d**) exhibited a marked inflammatory response with extensive submucosal oedema (O), fibrin deposits and polymorphonuclear inflammatory cells in the submucosa (not visible). The epithelium was sloughing and becoming detached from the submucosa in many places (E). Necrotic fat adhered to the outer wall of the bladder (N). **e,f**: Control specimens of kidney and bladder from an untreated mouse (same magnification as **a** to **d**).

neoplastic lesions, are probably required for effective tumour control. Permanent reductions in bladder capacity necessitating cystectomy are rare but can occur, particularly if PDT is given within 4 weeks of TUR, or if high total light doses are given (Harty *et al.*, 1989; D'Hallewin *et al.*, 1992).

The biological effect of PDT is determined by the energy absorbed by the photosensitiser. For a constant light dose, effect is determined by the tissue drug concentration, which depends on both administered dose and the time interval allowed before illumination. The rationale for delaying illumination for 48–72 h after administration of haematoporphyrin derivative sensitisers is that these compounds are cleared less rapidly from tumours than normal tissues and that a better therapeutic ratio is obtained by delaying illumination. Recent experiments of Baumgartner *et al.* (1992), for example, demonstrated similar levels of Photofrin in chemically induced rat bladder tumours and the surrounding normal bladder at 24 h after injection, but a 2 to 5-fold higher drug level in the tumour at 2 to 10 days. Detectable levels of the fluorescent component of Photofrin were, however, present in the normal bladder for up to 10 days. Drug uptake and distribution studies in mice have also demonstrated a slow clearance of these sensitisers from other normal tissues (Peng *et al.*, 1991; Bellnier *et al.*, 1989), with detectable drug levels in muscle, skin, heart, lung, spleen, kidney and liver at 75 days after injection of 5 mg $kg^{-1}$ Photofrin (Bellnier *et al.*, 1989). Several studies have also demonstrated longer elimination times for Photofrin (plasma and tissue) after i.p. administration than after i.v. administration, although absolute drug concentrations were higher in some organs (liver and kidney) after i.v. administration (Bellnier *et al.*, 1989; Peng *et al.*, 1991). Photofrin levels in the bladder were not measured in these studies. We have never examined the bladder response to PDT after i.v. administration of Photofrin and it is possible that route of injection may affect the extent of damage. Further studies will be required to clarify this issue.

The present studies demonstrate that there was no decrease in the extent of photosensitisation in normal mouse bladder for light applied at 1 to 7 days after 10 mg kg$^{-1}$ Photofrin. This would have the practical consequence that any unplanned delay in illumination would not be expected to reduce the efficacy of treatment, provided that illumination occurred within 1 week. It may also be possible to re-illuminate the bladder at any time during the first week, without the need for additional photosensitiser, if repeated therapy was judged to be necessary. This would, however, only be true if photobleaching (from the first illumination) had not reduced sensitiser levels in the tumour to below a therapeutic level. Interestingly, there was no evidence for increased bladder damage when the interval between drug an illumination was reduced to 6 h. It is not known whether i.v. administration of Photofrin would have lead to an increased response at 6 h.

Doses of 5 to 10 mg kg$^{-1}$ Photofrin at 1 day before illumination all caused significant increases in urination frequency and haematuria during the first 4 weeks. Histology confirmed that the functional damage induced by 5 or 10 mg kg$^{-1}$ was associated with a moderate to severe inflammatory response (in the mucosal and submucosal layers), with submucosal oedema and epithelial sloughing in all cases. Re-epithelialisation appeared to be almost complete within 4 weeks. The kidneys of PDT treated animals were also routinely taken for histology after sacrifice at 1 year and an occasional ($<$10%) incidence of pyelonephritis was seen. In one case this was associated with very severe bladder damage with bacterial infection and ascending urinary tract infection may have been the cause of the renal damage. There were, however, a few mice with severe renal damage where the bladders appeared normal. In these mice, transient ureteral sclerosis, probably caused by inflammatory changes during the acute reaction, may have impeded the normal flow of urine and contributed to an ascending urinary tract infection. It is unlikely that the renal damage observed in these studies occurred as a direct result of phototoxicity in the kidney, since the light dose (measured *in situ*) at the position of the kidney was less than 10% of the bladder dose. It is of interest that hydronephrosis has also occasionally been see in in patients treated with PDT of the bladder (Harty *et al.*, 1989).

An optimal photodynamic treatment for whole bladder could be defined as a combination of light and drug doses which gives a moderately severe acute response, with inflammation and epithelial sloughing, and complete healing over a period of a few weeks. Our studies would indicate that, for the mouse bladder, a dose of 5 to 10 mg kg$^{-1}$ Photofrin, given 1 to 7 days before whole bladder illumination with an incident, non scattered light dose of 11.3 J cm$^{-2}$ achieves this aim. The total fluence, including scattered light, in a closed system like the bladder is always much greater than the incident light dose (Star *et al.*, 1987) but can only be determined accurately if the optical properties of the tissue in question are known. Previous calculations (see discussion of Stewart *et al.*, 1992) indicate that the total fluence in these mouse bladders is three to four times the incident light dose. The bladder is able to recover completely from PDT using such schedules. However, care should be taken to avoid bacterial infection during the acute reaction period, since ascending urinary tract infection may well be associated with permanent renal damage.

We are very grateful to Lederle, Etten-Leur, The Netherlands for supplying the Photofrin and to Dr Brian Henderson, Heriot-Watt University, Edinburgh for making the diffusing light bulb fibres. We would also like to thank Drs P. Baas, N. Van Zandwijk, J. Marijnissen and W. Mooi for many fruitful discussions throughout this project and Miss Thea Eggenhuizen for preparation of this manuscript. This work was financially supported by the Dutch Cancer Society, project NKI 90-08.

### References

BAUMGARTNER, R., FUCHS, N., JOCHAM, D., STEPP, H. & UNSOLD, E. (1992). Pharmacokinetics of fluorescent polyporphyrin Photofrin II in normal rat tissue and rat bladder tumor. *Photochem. Photobiol.*, **55**, 569–574.

BELLNIER, D.A., HO, Y.-K., PANDEY, R.K., MISSERT, J.R. & DOUGHERTY, T.J. (1989). Distribution and elimination of Photofrin II in mice. *Photochem. Photobiol.*, **50**, 221–228.

BENSON, R.C. (1985). Hematoporphyrin photodynamic therapy for transitional cell carcinoma of the bladder – an update. *Adv. Exp. Biol. Med.*, **193**, 3–12.

D'HALLEWIN, M.A., BAERT, L., MARIJNISSEN, J.P.A. & STAR, W.M. (1992). Whole bladder wall photodynamic therapy with in situ light dosimetry for carcinoma *in situ* of the bladder. *J. Urology*, **148**, 1152–1155.

EDREES, G., LUTS, A. & STEWART, F. (1988). Bladder damage in mice after combined treatment with cyclophosphamide and X rays. The influence of timing and sequence. *Radiother. Oncol.*, **11**, 349–360.

HARTY, J.I., AMIN, M., WIEMAN, T.J., TSENG, M.T., ACKERMAN, D. & BROGHAMER, W. (1989). Complications of whole bladder dihematoporphyrin ether photodynamic therapy. *J. Urology*, **141**, 1341–1346.

HISAZUMI, H., MISAKI, T. & MIYOSHI, N. (1983). Photoradiation therapy of bladder tumors. *J. Urology*, **130**, 685–687.

JOCHAM, D. (1987). Photodynamic techniques in the treatment of bladder cancer. In *Advance in Urologic Oncology. Vol. 1. General Perspectives*. Williams, R.D. (ed.), pp. 111. Macmillan Pub Co: New York.

MARIJNISSEN, J.P.A., STAR, W.M., IN 'T ZAND, H.J.A., D'HALLEWIN, M.A. & BAERT, L. (1993). *In situ* light dosimetry during whole bladder wall photodynamic therapy: clinical results and experimental verification. *Phys. Med. Biol.*, **38**, 1–16.

NAITO, K., HISAZUMI, H., UCHIBAYASHI, T., AMANO, T., HIRATA, A., KOMATSU, K., ISHIDA, T. & MIYOSHI, N. (1991). Integral laser photodynamic treatment of refractory multifocal bladder tumurs. *J. Urology*, **146**, 1541–1545.

NSEYO, U.O., DOUGHERTY, T.J. & SULLIVAN, L. (1987). Photodynamic therapy in the management of resistant lower urinary tract carcinoma. *Cancer*, **60**, 3113–3119.

PENG, Q., MOAN, J., KONGSHAUG, M., EVENSEN, J.F., ANHOLT, H. & RIMINGTON, C. (1991). Sensitizer for photodynamic therapy of cancer: a comparison of the tissue distribution of photofrin II and aluminium phthalocyanine tetrasulfonate in nude mice bearing a human malignant tumor. *Int. J. Cancer*, **48**, 258–264.

POPE, A.J. & BOWN, S.G. (1991). The morphological and functional changes in rat bladder following photodynamic therapy with phthalocyanine photosensitization. *J. Urology*, **145**, 1064–1070.

PROUT, G.R., LIN, C.-W., BENSON, R., NSEYO, U.O., DALY, J.J., GRIFFIN, P.P., KINSEY, J., TIAN, M.-E., LAO, Y.-H., MIAN, Y.-Z., CHEN, X., REN, F.-M. & QIAO, S.-J. (1987). Photodynamic therapy with hematoporphyrin derivative in the treatment of superficial transitional-cell carcinoma of the bladder. *N. Engl. J. Med.*, **317**, 1251–1255.

SHUMAKER, B.P. & HETZEL, F.W. (1987). Clinical laser Photodynamic therapy in the treatment of bladder carcinoma. *Photochem. Photobiol.*, **46**, 899–901.

SINDELAR, W.F., DELANEY, T.F., TOCHNER, Z., THOMAS, G.F., DACHOWSKI, L.J., SMITH, P.D., FRIAUF, W.S., COLE, J.W. & GLATSTEIN, E. (1991). Technique of photodynamic therapy for disseminated intraperitoneal malignant neoplasms. Phase I study. *Arch. Surg.*, **126**, 318–324.

STAR, W.M., MARIJNISSEN, H.P.A., JANSEN, H., KEIJZER, M. & VAN GEMERT, M.J.C. (1987). Light dosimetry for photodynamic therapy by whole bladder wall irradiation. *Photochem. Photobiol.*, **46**, 619–624.

STEWART, F.A., MICHAEL, B.D. & DENEKAMP, J. (1978). Late radiation damage in the mouse bladder as measured by increased urination frequency. *Radiat. Res.*, **75**, 649–659.

STEWART, F.A., OUSSOREN, Y., TE POELE, J.A.M., HORENBLAS, S. & MOOI, W.J. (1992). Functional and histological damage in the mouse bladder after photodynamic therapy. *Br. J. Cancer*, **65**, 884–890.